# MIA-NDN: Microservice-Centric Interest Aggregation in Named Data Networking

**DOI:** 10.3390/s23031411

**Published:** 2023-01-27

**Authors:** Muhammad Imran, Muhammad Salah Ud Din, Muhammad Atif Ur Rehman, Byung-Seo Kim

**Affiliations:** 1 Department of Software and Communications Engineering, Hongik University, Sejong City 30016, Republic of Korea; 2 Department of Electronics & Computer Engineering, Hongik University, Sejong City 30016, Republic of Korea; 3 Department of Computing & Mathematics, Manchester Metropolitan University, Manchester M15 6BH, UK

**Keywords:** microservice interest aggregation, information-centric networking, named data networking, pending interest table, microservice-centric computations

## Abstract

The named data networking (NDN)-based microservice-centric in-network computation poses various challenges in terms of interest aggregation and pending interest table (PIT) lifetime management. A same-named microservice-centric interest packet may have a different number of input parameters with nonidentical input values. In addition, the same-named interest packet with the same number of parameters may have different corresponding parameter values. The vanilla NDN request aggregation (based on the interest name, while ignoring the input parameters count and/or their corresponding values) may result in false aggregation. Moreover, the microservice-centric requested computations may fail to accomplish in the default 4s PIT timer due to the input size. To address these challenges, this paper presents MIA-NDN: microservice-centric interest aggregation in named data networking. We designed microservice-centric interest-naming to enable name-based communication. MIA-NDN develops a robust interest aggregation mechanism that not only performs the interest aggregation based on the interest name but also considers the input parameter counts and their corresponding values in the interest aggregation process to avoid false packet aggregations. A dynamic PIT timer mechanism based on input size was devised that avoids the PIT entry losses if the execution time exceeds the default PIT timer value to avoid computation losses and uphold the application quality of service (QoS). Extensive software-based simulations confirm that the MIA-NDN outperforms the benchmark scheme in terms of microservice-centric interest aggregation, microservice satisfaction rate, and communication overhead.

## 1. Introduction and Motivation

The advancements in Internet of Things (IoT) technologies and communication, computation, and sensing capabilities of smart devices have led to the foundation of several intelligent and ubiquitous services [1,2]. These devices enable massive content gathering and content dissemination through a variety of applications (i.e., delay tolerant and delay-sensitive) such as autonomous driving, smart health monitoring, video downloading, disaster monitoring, and social networking, to name a few [3]. IoT devices are usually resource-constrained, performing specific computations over the acquired data [4]; most of the conventional applications usually transfer their data to the central cloud station, which is usually located far away from the consumer. Therefore, transferring the data to the cloud may result in large latency, network congestion, and excessive bandwidth utilization. Moreover, taking delay-intolerant applications into consideration, the aforementioned consequences may severely affect the on-time decision capabilities of the application, which may result in disastrous consequences [5].

To handle the cloud deficiencies, edge computing has emerged as a promising candidate with the precise motivation to bring the cloud facilities (such as computing, storage, and networking resources) closer to the consumer [6,7]. Edge computing provides large computations at the network edge in the closed vicinity of the user with reduced bandwidth consumption and fewer delays, minimizing the chances of congestion in the network [8,9]. Edge computing undeniably provides a resourceful platform for these applications to accomplish their computations within the strict latency requirements, however, peak workload conditions (e.g., a burst of compute-intensive consumer requests) may over-utilize the edge resources. To avoid computation loss, edge terminals offload the requested computation toward the cloud [10], which turns in performance degradation, bandwidth utilization, excessive communication delays, location-less communications, content dissemination, content security, and high consumer and producer mobility [11].

Currently, communication between IoT, edge, and cloud is carried out through conventional address-centric communication mechanisms (i.e., TCP/IP) where an end-to-end communication path is required to be established before initiating the communication [12]. With the evolution in technology and the development of data-centric applications, the current internet operation is changing from a host-centric to a content-centric model [13], meaning that current user applications mainly focus on the required content regardless of the originator’s physical location [14]. Instead of reaching the actual content producer, any networking device residing within the network with the requested content in its storage must be able to respond with the data to uphold the QoS and optimize the networking resources [15]. The current IP architecture lacks the aforementioned functionalities.

Modern applications, such as augmented reality (AR)/virtual reality (VR)-based systems, the metaverse, and the IoT mostly require content irrespective of the originator’s location for efficient operation. However, conventional IP-based communication necessitates the establishment of an end-to-end communication connection between the consumer and producer to initiate communication, meaning that the consumer’s request must have to reach the producer to fetch the content irrespective of the availability of the requested content on the intermediate computing or networking devices, which results in high latency and excessive bandwidth consumption. To handle the mismatch between the conventional IP-based internet architecture and application requirements, information-centric networking (ICN) [16] and flavor-named data networking (NDN) [17] have emerged as potential candidates for the future internet architecture. Irrespective of host-centric centric IP networking, NDN breaks the connection-oriented communication philosophy and considers the content as first-class citizens. The NDN consumers only specify what they require irrespective of where the content is located. The data are self-authenticating and can be acquired by the consumer via application-specific, location-oblivious, unique, and persistent names. In addition, ICN’s enchanting features, such as request aggregation (using pending interest table (PIT)), stateful forwarding (via FIB), and in-network caching (CS) promote efficient content delivery and reduce data retrieval delays [18].

Taking the NDN-based microservice-centric in-network computations into account, the vanilla NDN may face several performance issues in terms of request aggregation and PIT lifetime management. The vanilla NDN performs the PIT interest aggregation based on the interest name; the default lifetime (i.e., 4 s) is allocated to each PIT entry. With microservice-based interest names, multiple interests may correspond to the same name; however, they may have (1) the same number of the input parameters but different parameter values, and (2) a different number of input parameters. Taking the aforementioned microservice input parameters heterogeneity, the vanilla NDN may never perform accurate interest aggregation. Moreover, the requested computations may never accomplish (i.e., in the default, 4 s) the PIT timer due to the input size. These factors may result in false aggregation, packet losses, long latencies, and excessive network resource utilization.

Researchers have focused on different state-of-the-art schemes [19,20,21] in the literature, mainly targeting effective in-network service computations. The scheme presented in [19] sends a computing request to the producer and the producer estimates the request execution time, generates the acknowledgment packet, and sends it back to the consumer to adjust the PIT lifetime and maintain the entry of intermediate nodes. A similar scheme proposed in [20] first inquires about the potential function executor by forwarding computing interests and generating a separate interest packet to carry the remote method invocation parameters toward the potential forwarder, maintaining a 5 s static PIT lifetime for computation request entries. Another service execution scheme named "serving at the edge” presented in [21] forwards the interest toward the compute node. The compute node informs the consumer about the execution via an acknowledgment packet and pushes the results back toward the consumer after generating the results. These schemes mainly focus on in-network computation-based PIT lifetime management; however, these schemes ignore the input parameters and their corresponding values of microservice requests. Therefore, these schemes lack efficient PIT lifetime management mechanisms based on microservice input sizes (i.e., the small input may take lower computation times and deliver the results in minimum times compared with large-sized input).

To address the above-mentioned challenges, this paper extends the current NDN architecture and proposes a robust microservice-centric interest aggregation and PIT lifetime management mechanism named MIA-NDN. MIA-NDN takes the input parameters as well as their corresponding values into account in its interest aggregation process. MIA-NDN devised tailored naming schemes comprised of microservice names and input parameters. A dynamic PIT lifetime management mechanism is provided that considers microservice computations and communication costs. In addition, MIA-NDN computes the hash value of each interest packet to avoid false interest aggregation in the PIT table.

In summary, the following are our core contributions to the proposed scheme.

1. We extend the vanilla NDN architecture to support the microservice-based in-network computations by proposing a state-of-the-art microservice-centric interest-naming mechanism that incorporates the content name, microservice name, input parameters, and delimiters for separating the multiple components.
2. MIA-NDN developed a dynamic PIT timer based on microservice input parameters and their corresponding input values to avoid PIT entry losses in the event of long-running microservice computation interests.
3. A hash-based PIT aggregation mechanism was developed to achieve efficient microservice-centric PIT aggregation, taking into consideration the input parameters and their corresponding values to make every entry unique in the PIT table.
4. MIA-NDN was evaluated based on extensive NDNSim-based simulations to reveal the potential benefits in terms of efficient interest aggregation, microservice computation satisfaction, and network overhead.

The remainder of the paper is organized as follows. Section 2 presents the background and related work. Section 3 describes the proposed scheme. Paper evaluation and simulation results are discussed in Section 4. Finally, Section 5 concludes the paper and presents future work.

## 2. Background and Related Work

In this section first, we present the overview of the microservices and NDN, and then we provide the related work.

### 2.1. Background

**Figure 1:**
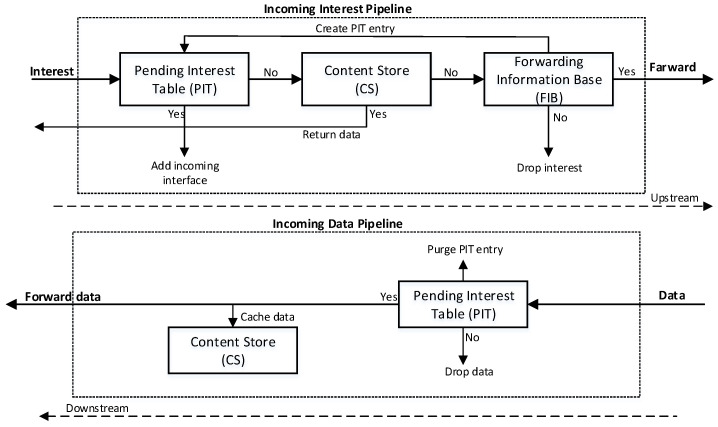
NDN Communication Process.

1. **Microservices in a nutshell:** Microservices are gaining more attention from enterprises. Big tech companies, such as Netflix, Twitter, Amazon, and Spotify utilize microservices in their businesses [22]. Microservices are proposed software development architectures used to create applications as loosely coupled small components [23]. Such small components are easy to develop, deploy, and test independently. Each component performs its own task and communicates with other microservices through well-defined communication interface. The small components feature enables microservice scalability, allowing one to update and change a component without affecting other components [24].
2. **Named data networking in a nutshell:** NDN internet architecture is a shift from host-centric to data-centric communication and provides named content-based communication [17]. In NDN, two types of packets interests and data are used in communications. NDN allows consumers to send content-named interests and retrieve corresponding data packets at the network layer. A detailed process of the NDN interests and data packets is illustrated in Figure 1. Each NDN router maintains three tables. (1) Content store (CS): CS is transient storage space at the NDN router that stores the copy of incoming data packets to satisfy future consumer-generated requests for the same data. (2) Pending interest table (PIT): The PIT stores the entries of forwarded interests that are waiting for the required data. Each PIT entry waits for the data packet until its associated timer value. (3) Forwarding information base (FIB): FIB keeps the information from the content producer or provider.

### 2.2. Related Work

NDN is recognized as an in-network computation enabler [25,26], where named services [19,27] and named functions perform the consumers’ requested content computations. In the literature, several approaches are proposed that consider performing the computations in the NDN network.

The pioneering work, named function as a network (NFN) [25], enables data processing in the NDN network. In NFN, a name represents the mapping to the content and the function to content processing; expressions represent the combination of both the content name and function. The interest packet has named data as well as function information, and the network is responsible for discovering an executing node and expression resolution with name-based forwarding to execute a function. A general-purpose framework named function service (NFaaS) is similar to NFN as it extends the NDN architecture to support in-network function computations [28]. In NFaaS, the computation functions are provided by utilizing virtual machines (VMs) instead of lambda expressions, as presented in [25]. The VM-hosted kernel stores the function codes and makes decisions on which functions to download and execute locally. The functions can also migrate toward the edge according to the function’s demands and the function demands can depend on delay-sensitive and bandwidth-hungry applications. For delay-sensitive applications (AR/VR, autonomous vehicles), the function migrates toward the data-generating source to meet strict delays, and the bandwidth-hungry application runs close to the network core, remaining within the edge boundaries.

A similar work was proposed by the authors of [29], in which the authors designed a computation service management (CS-Man) protocol by employing NFN, which enables the IoT tasks in-network processing based on the device’s current workload status. The CS-Man works in two modules; the first is the service discovery, which is used to obtain network information about the capable nodes, which can provide services, and the second one is service deployment, which deploys the interests to the appropriate candidate nodes for execution. The CS-Man discovers the services and deploys tasks according to the workload conditions; consequently, it lowers the network traffic and achieves the computations efficiently. A dynamic computing environment for IoT data processing is proposed in [30], which makes data retrieval possible as well as data processing at the edge of the network. In this scheme, the authors defined naming and forwarding strategies, and these strategies guide the requests for service toward edge computing nodes that are very close to data sources in order to limit the raw data transmission in the network. The proposed scheme achieves lower volumes of data communications and service delivery times.

A keyword-based naming scheme for IoT retrieval and local computation was proposed in [31]. The proposed scheme comprises (i) a hierarchical name prefix that allows the routing to locate the IoT processing, and (ii) a keyword as a suffix to indicate specific data in the IoT domain. The hierarchical name prefix finds the route toward the IoT domain and exploits available edge resources for computations. In this scheme, interest carries the function name and information about how the processing should be done; the producer only returns a single data item. The proposed scheme was evaluated on three different strategies for locating and processing IoT data with the IoT edge domain; it was found that the scheme outperforms in terms of latency and reduces network overhead. The authors of [32] proposed Hydro—a hybrid function orchestration scheme for distributed computing in information-centric networking. The Hydro scheme employs a logically centralized coordinator that gathers the compute node’s computation information in order to reduce the function execution time and balance resource utilization. It collects information on function deployment and network resource utilization to push computations from the cloud to underutilized edge nodes closer to users. The simulation results were compared with NFN; the results revealed that Hydro outperforms and improves computation completion times (to 51%).

Lia et al. [33] proposed an in-network task placement strategy that aims to minimize network resource utilization. This strategy achieves task allocation to the proximal edge nodes, minimizing the execution latency. The results of the proposed strategy are compared against a cloud-only state-of-the-art solution and the results prove that the proposed scheme outperforms. A unified remote method invocation (RICE) proposed by the authors in [20] exploits ICN properties, such as name-based routing, receiver-based congestion control, flow balance, and object security. RICE is a network layer general-purpose framework that can be applied to any named function networking. RICE employs the thunks [34], a concept of a programming language to decouple the method invocation from the return of long-running application results. The ultimate goal of RICE involves in-network function execution, consumer authentication, and non-trivial parameter passing. It supports scenarios where computations take longer than the default PIT expiry times.

To discover the in-network compute nodes in the network, the authors of [35] presented DiCer: a distributed coordination for in-network computations strategy. DiCer adopts the state vector synchronization (SVS) dataset protocol in ICN to increase the neighborhood information of compute nodes in a distributed manner. DiCer assists in service deployment by increasing the resolution of computing requests. The DiCer scheme was evaluated against NFN and it was found that it increases resource utilization at the edge and reduces the request completion time. A service executing at the edge architecture based on ICN is presented by the authors of [25]. This architecture contains the edge computing service session model, requesting forwarding strategies, and dynamic service deployment mechanism. This scheme attempts to keep the overhead low by pushing the computation results toward the consumer immediately upon the computation completion. The simulation results indicate that the proposed scheme lowers a state communication overhead, and the forwarding strategies achieve service completion times with a low probing overhead.

In summary, the above-presented schemes do not consider microservice-centric interest-input parameters with their corresponding values in PIT aggregation and allocate the static PIT lifetime. Contrary to the existing solutions, in this paper, we propose a microservice-centric interest aggregation, PIT dynamic timer calculation, which has not been fully investigated yet. Specifically, we consider the same-named microservice-centric interests with different input parameters and the same-named microservice interests with the same number of input parameters but different corresponding values and dynamic PIT lifetime management based on interest parameter values to achieve true microservice-centric interest aggregation and computations efficiently.

## 3. Proposed Scheme

In this section, we provide the details of the proposed MIA-NDN scheme. First, we provide the overview of the MIA-NDN and then the technical details of the scheme.

### 3.1. Proposed Scheme Architecture

**Figure 2:**
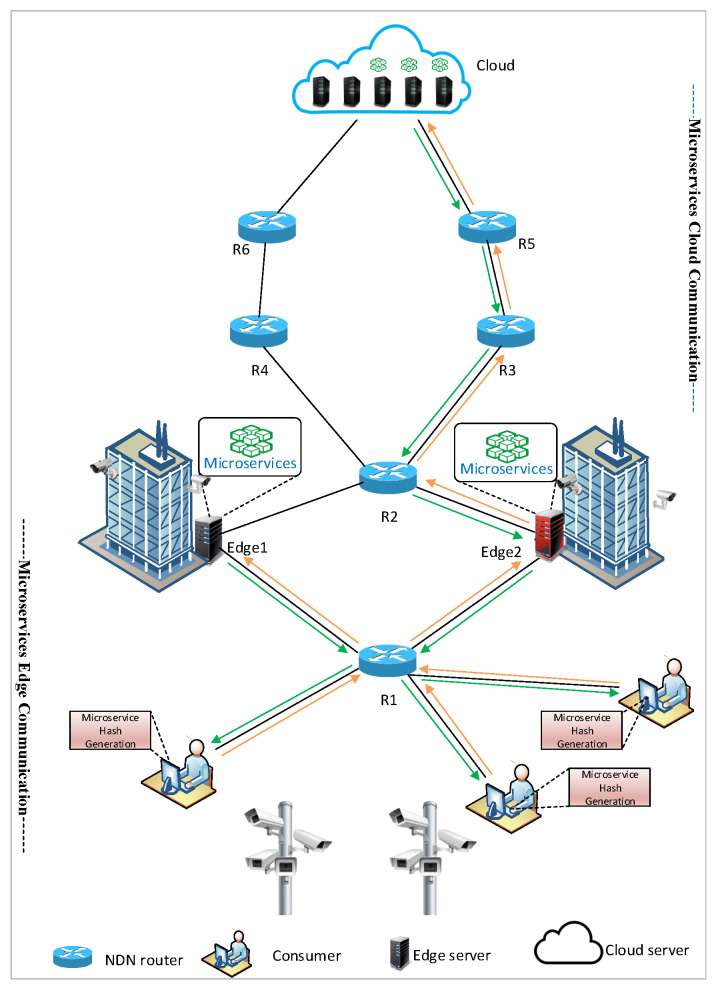
Proposed scheme architecture.

The overall architecture of the MIA-NDN scheme is presented in Figure 2. As shown in the figure, the consumer sends a microservice computation request to the edge node by generating and storing the hash value of a microservice interest in the interest packet (detailed in Section 3.2). After receiving the interest, the edge node performs the computation and returns the result to the consumer. The edge nodes may become overloaded due to multiple microservice requests; in that case, they eventually offload the computations toward the cloud (as Edge-2 shows in Figure 2). The edge node, prior to interest offloading, calculates the interest PIT lifetime (detailed in Section 3.3) and forwards interest toward the cloud. When forwarding the interest packet toward the cloud, the edge node sends an interest packet toward downstream routers and updates the PIT timers (e.g., nodes R1 and consumer NFD). We provide the details of the microservice packet format, interest hashing, and aggregation (Section 3.2), and the dynamic PIT timer calculations (Section 3.3).

### 3.2. Proposed Scheme

In this subsection, we provide the key details of the proposed scheme, such as the interest packet format, interest aggregation, and microservice-centric interest hashing.

**Figure 3:**
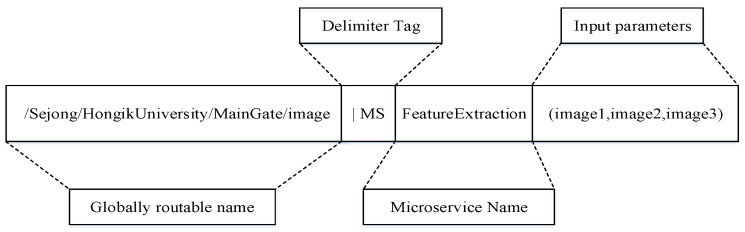
Microservice-centric interest packet structure.

**Figure 4:**
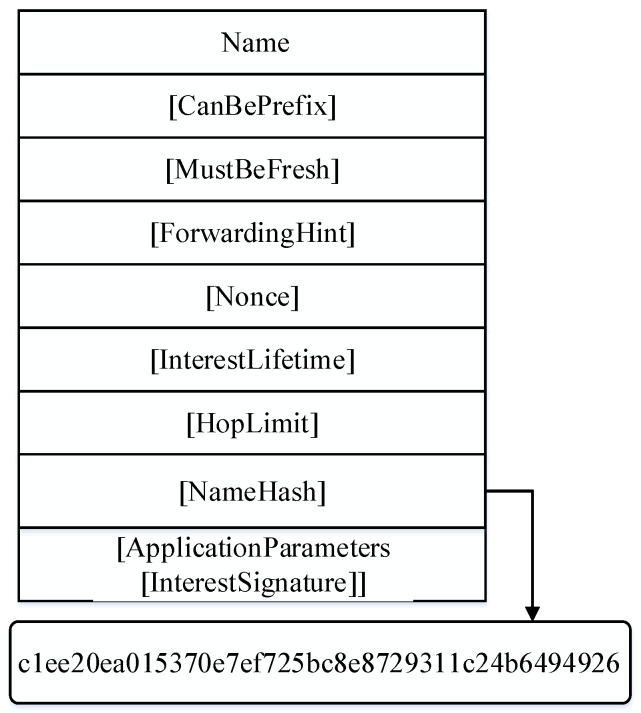
NameHash-based interest packet.

1. **Interest Packet Format:** In the proposed MIA-NDN scheme, the interest can request a microservices computation or simple content. When requesting simple content, the interest packet follows the conventional NDN content-naming structure. However, when requesting the microservice-centric content, the interest packet pursues the following microservice-centric interest-naming format as shown in Figure 3.
  In the MIA-NDN scheme, each interest is composed of a content name followed by a microservice name and input parameters. The microservice-centric interest packet has four parts, (i) the content name, (ii) the microservice tag, (iii) the microservice name, and (iv) input parameters. Figure 3 depicts the microservice-centric interest packet structure where the */Sejong/HongikUniversity/MainGate/image* represents the content name or globally routable name, *MS* is used as a delimiter tag to separate the microservice component from the content name, *FeatureExtraction* is the name of a microservice, and *image1, image2, image3* are the input parameters. Among the aforementioned name components, the first component is mandatory, whereas the last three components are optional.
2. **Interest Aggregation:** In the proposed MIA-NDN, the interest aggregation is comprised of two steps (i) the hashing and (ii) aggregation. At first, the microservice interest packet’s hash is calculated, after that, the interest aggregation is performed in the PIT table along with the hash value.
  A detailed description of the hashing and aggregation process is given as follows.
  (a) **Microservice-centric interest hashing and aggregation:** NDN’s core feature is content-naming, and it has a profound impact on network performance (e.g., lookup and memory consumption). microservice-centric interests may have large-sized interest packets, for example in the feature extraction scenario where input parameters may contain large-sized images, and such packets may consume a considerable amount of memory in the PIT table.
    The NDN is a search-based internet architecture, where tables (CS, PIT, and FIB) are consulted before interest and data packet forwarding; therefore, such large-size interest aggregation in the PIT table is not an optimal solution.
    The aggregation of such large-sized interests in the PIT table may exhaust the NDN node’s memory.
    In addition, the PIT table lookup is required for finding similar interests that have already been forwarded and are waiting for results to perform the same incoming request aggregation. Such large-sized interest-matching in the PIT table requires a high search time, which ultimately degrades the network performance. Therefore, in microservice-centric interest aggregation, the PIT table’s memory consumption, searching time, and searching costs are required to be optimized.
    To resolve the above-mentioned issues, the proposed MIA-NDN scheme computes a hash value of microservice interests and stores it in the PIT table.
    For hashing, the proposed scheme employs the SHA-256 hashing algorithm that generates the hash value of the incoming interest packet’s name components (i.e., content name, microservice name, and input parameters) after concatenating them together.
    The SHA-256 algorithm generates a 32-bit hash value, which is efficient to store in the PIT table instead of storing several megabytes of microservice parameter interests. The hash value is computed as soon as the networking forwarding daemon (NFD) of a consumer node receives an interest packet. The consumer then creates a unique PIT entry by storing the hash value as a content name. Finally, after updating the outgoing interface of a PIT entry and before forwarding the packet toward the upstream node, the computed hash value is stored inside the [NameHash] field of an interest packet as depicted in Figure 4. The rationale for storing the hash value is to avoid microservice interest false aggregation, optimum PIT table memory consumption, PIT searching time, and searching cost minimization. In the latter incoming interests, the stored hash value is compared for interest aggregation purposes. In case a hash match is found, the aggregation is performed, otherwise, a new PIT entry is created, and interest is forwarded to the provider/producer by following the FIB entry.

### 3.3. Dynamic PIT Timer

The microservice executions might be compute-intensive tasks, especially in the case of image processing and feature extraction microservices; therefore, the vanilla NDN default PIT timer (4 s) may not be adequate to complete microservice computations and yield the results on time. Therefore, the objective is to develop a dynamic PIT timer that adjusts the PIT entry lifetime according to the microservice parameter count and the respective size, enabling the intermediate nodes to maintain the PIT entry for a sufficient time. In that way, the proposed scheme avoids the computation results from losses and the over-utilization of network resources that may occur due to default (static) PIT timer settings.

**Figure 5:**
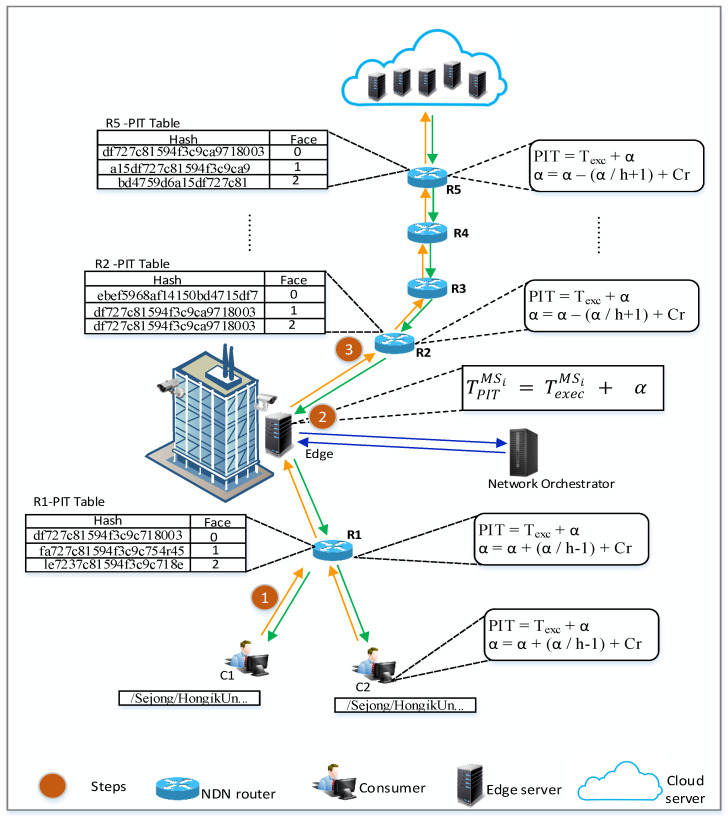
Dynamic PIT timer calculation.

In the proposed scheme, the dynamic PIT timer on the intermediate nodes is calculated by analyzing the (i) microservice interest packet and (ii) the communication cost between the edge node and the executing node. If the size of a microservice parameter is large (in terms of bytes), the large PIT timer value is selected, otherwise, a short PIT timer is selected in case the parameter value size is small. Employing such dynamicity in the PIT timer setting by considering the interest parameter size enables network packet processing within the pending lifetime as well as optimizes the PIT table size. In addition to the microservice parameters, the PIT timer calculation is also based on the communication cost, i.e., the time it takes to offload a microservice request from a consumer to reach the cloud server and the results to receive back. The edge node consults the network orchestrator (detailed in the next subsection), which keeps a record of the hops data between two ends and the computation resource utilization status of the cloud server (**Assumption**. The interest forwarding path is known; in Figure 5, R2 knows that it has to forward the interest packet to R3; R3 to R4; and so on. It is based on the FIB, which is out of the scope of our work).

In the following, we provide a detailed description of the working mechanism of the network orchestrator, explaining how it assists the dynamic PIT timer calculation in microservice computational offloading.

**Figure 6:**
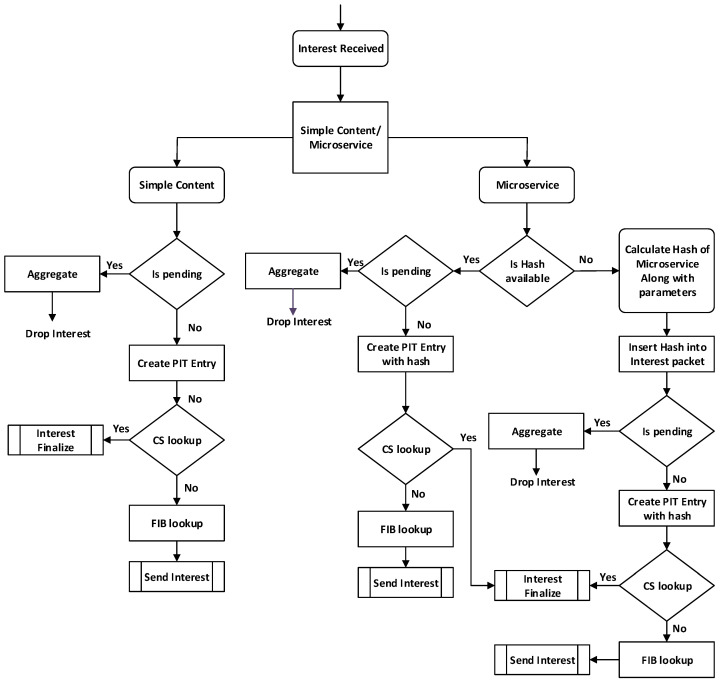
The microservice-centric interest processing pipeline.

1. **The Role of the Network Orchestrator:** A network orchestrator is a network management node that keeps network topology and cloud node computation load information. As shown in Figure 5, consumers C1 and C2 send a microservice request for the computations and it is received at R1 (e.g., in the figure, the yellow arrow represents interests, and the green arrow represents data packets). Then, R1 forwards the request toward the edge node. The edge node may not have enough resources to execute the microservice request. Therefore, the edge node offloads the request to the cloud server. However, before offloading a request, the edge node consults the network orchestrator by sending the consumer’s received microservice interest packet to obtain the information about (i) the communication time required to take the data packet from sending an interest packet and, (ii) the computation time required to perform the microservice computation on a compute node.
  The network orchestrator node calculates the computation time based on the cloud server’s load status and the required resources of the requested microservice interest packet (the network orchestrator forwards the request to the light-loaded cloud node). After calculating the computation time, the network orchestrator node sends the computation time and communication time (e.g, data of the intermediate nodes) by storing them inside the interest packet back to the edge node. Therefore, the edge node based on the communication time and computation time calculates and sets its PIT timer and offloads the interest packet toward the cloud by storing intermediate nodes and computation time information in the interest packet in step 2 (the intermediate nodes and computation time information is shared with upstream nodes to avoid contacting the network orchestrator node all of the time). R2, upon receiving the interest, calculates its PIT timer based on the computation time and communication time information obtained from the interest packet.
  The computation time remains constant for all intermediate nodes while the communication time decreases gradually as interest approaches the cloud server. Therefore, the intermediate nodes calculate the communication time based on the hop distance and set their PIT timers accordingly (step 3). Finally, at the time of interest offloading, the edge node sends an interest packet toward the downstream nodes as well as the PIT timer update to avoid the pending entry earlier timeouts. Consequently, R1 and consumers calculate and update their PIT timers of the microservices’ pending interests.
2. **Dynamic PIT Timer Calculation:** The edge and intermediate nodes calculate their PIT timers dynamically based on the network orchestrator’s computation time and communication time information. The computation and communication time calculations are described as follows.
  Let $T_{PIT}^{MS_{i}}$ be the total PIT lifetime, including the communication time and computation time required to send an interest, performing the computation at the compute node, and receiving results (data packet) of the microservice interests $MS_{i}$ [36]. The $T_{PIT}^{MS_{i}}$ can be calculated by using the following equation:
  (1) $$T_{PIT}^{MS_{i}}=T_{exec}^{MS_{i}}+a$$
  where $T_{exec}^{MS_{i}}$ is the time required to execute an interest at a compute node and *a* is the total time required to send an interest and receive a data packet (communication time).
  The $T_{PIT}^{MS_{i}}$ is calculated based on the load status of a compute node obtained from the network orchestrator (Section 3.3). The microservice interest execution time can be calculated by the following equation:
  (2) $$T_{exec}^{MS_{i}}=\left\{\begin{array}{cc} \frac{MS_{sz}^{i}}{CPU_{avc}^{i}} & if\;MS_{sz}^{i}\;and\;CPU_{avc}^{i}>0\\ 0 & Otherwise \end{array}\right.$$
  The $MS_{sz}^{i}$ is the total size of the $i_{\mathrm{th}}$ microservice interest in bytes and $CPU_{avc}^{i}$ is the available CPU cycles on the $i_{\mathrm{th}}$ compute node where information is obtained from the network orchestrator. The $MS_{sz}^{i}$ is comprised of the microservice input parameters and their corresponding sizes. The computation time is calculated by adding all input parameter sizes and dividing by the available CPU cycles of the compute node.
  In Equation (1), *a* is the communication time required to send a microservice interest packet and receive a data packet. The *a* can be calculated (e.g., between the edge and cloud server) by the following equation:
  (3) a=a−(a/h+1)+cr
  where *h* represents the number of intermediate nodes from offloading the edge server to the cloud server and *cr* is a congestion rate of a link between the intermediate nodes. The FIB provides information about the congestion rate between nodes.
  The PIT timer on the downstream node (the edge to the consumer) is updated by the following equation.
  (4) a=a+(a/h−1)+cr
3. **Interest processing pipeline of the proposed scheme:** A detailed description of microservice-centric interest processing in the MIA-NDN scheme is given below with the help of a flow chart. In Figure 6, the microservice-centric interest processing steps are summarized. A detailed description of the steps is given as follows.
  (a) After receiving the interest packet, the edge node checks the packet type to determine whether the received interest is conventional NDN content or a microservice-centric request by searching the *MS* tag. In the presence of an *MS* tag, the interest packet is processed according to the microservice interest processing pipeline, otherwise, the interest packet is forwarded to the conventional NDN processing pipeline.
  (b) Once it is determined that the received interest packet contains a microservice request tag, the edge node then checks whether the NameHash field contains a value. In the presence of the NameHash value, the edge node searches the PIT entry with the NameHash value. In the absence of the NameHash value, the edge node performs the hash calculation according to step 4 and adds it to the interest packet.
  (c) In the presence of the NameHash value, the pending PIT entries are searched by comparing the hash value. If a hash match is found, the edge node performs the aggregation and drops the interest packet. In the absence of pending entry, the new PIT entry is created, the CS searches for the matching data (results) and is subsequently followed by the conventional interest processing steps. The results stored in the CS also contain a hash value along with the content name for the same future request fulfillment.
  (d) In the absence of a NameHash value, the edge node calculates the hash value after concatenating the content name, microservice name, and input parameter values using the SHA-256 hashing algorithm and inserts the obtained hash to the NameHash field before forwarding the interest packet.
  (e) After calculating and inserting the hash value of interest, the PIT table is consulted to check that the interest is pending. In case the interest is pending, the aggregation is performed, otherwise, the new PIT entry is created.
  (f) After the PIT, then CS lookup is performed to check the data availability in the router’s cache; if data are available in the cache, the interest is finalized and data are returned to the consumer, otherwise, the FIB is consulted, and interest is forwarded to the producer, otherwise, the packet is dropped.

## 4. Implementation

In this section, we describe the experimental environment, implementation, and evaluation of the proposed MIA-NDN scheme against the most recent existing solution [21].

### 4.1. Experimental Setup

To evaluate the effectiveness of MIA-NDN, we performed extensive simulations in NDNSim (an ns3-based simulator on a computer equipped with Core i5, 16 GB of RAM) and compared the results with the state-of-the-art scheme named Serving at the Edge (SATE) [21]. In our simulation environment, we consider 10 nodes, with 2 edges, 2 consumers, 6 NDN routers, and a cloud equipped with computation, communication, and storage units. To mimic the microservices behavior, we adopted the ndnCSIM (https://github.com/atifrehman/ndn-compute-simulator) (accessed on 20 November 2022) [37], codebase, where the necessary operations and requirements regarding microservice development and deployment were provided. For the extensive evaluation, we varied the microservice-based computation requests with (1) the same name, same parameters, and different parameter values, and (2) the same name and different input parameter counts at both high (i.e., 10 to 50 req/s) and low request rates (i.e., 1 to 10 req/s). The summary of our simulation setup is presented in Table 1.

The following performance metrics are considered for comparison evaluations.

1. **Interest aggregation:** Interest aggregation is defined as the total number of same-named microservice-centric interest packets aggregated to the total number of microservice interest packets transmitted.
2. **Microservices satisfaction rate:** The microservice interest satisfaction rate is the ratio of the total number of data packets received against the total number of microservice interest packets sent.
3. **Transmission overhead:** Transmission overhead measures the total number of packet transmissions (interest, acknowledgments, and data) in the network against the number of microservice computations.
4. **PIT density:** The PIT density is the ratio of the total number of microservice interests maintained in the PIT table to the total number of microservice interests generated in the network.

### 4.2. Simulation Results

**Figure 7:**
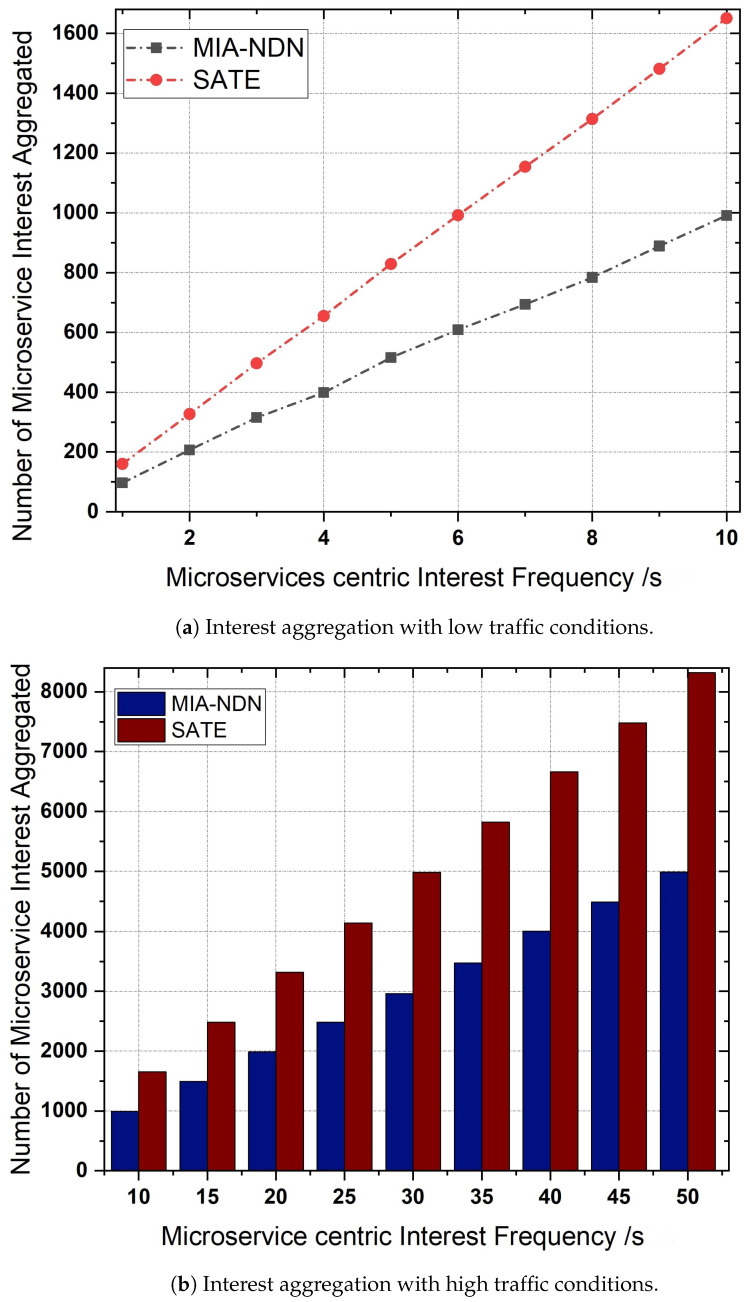
Microservice interest aggregation as a function of microservice interest frequency.

**Figure 8:**
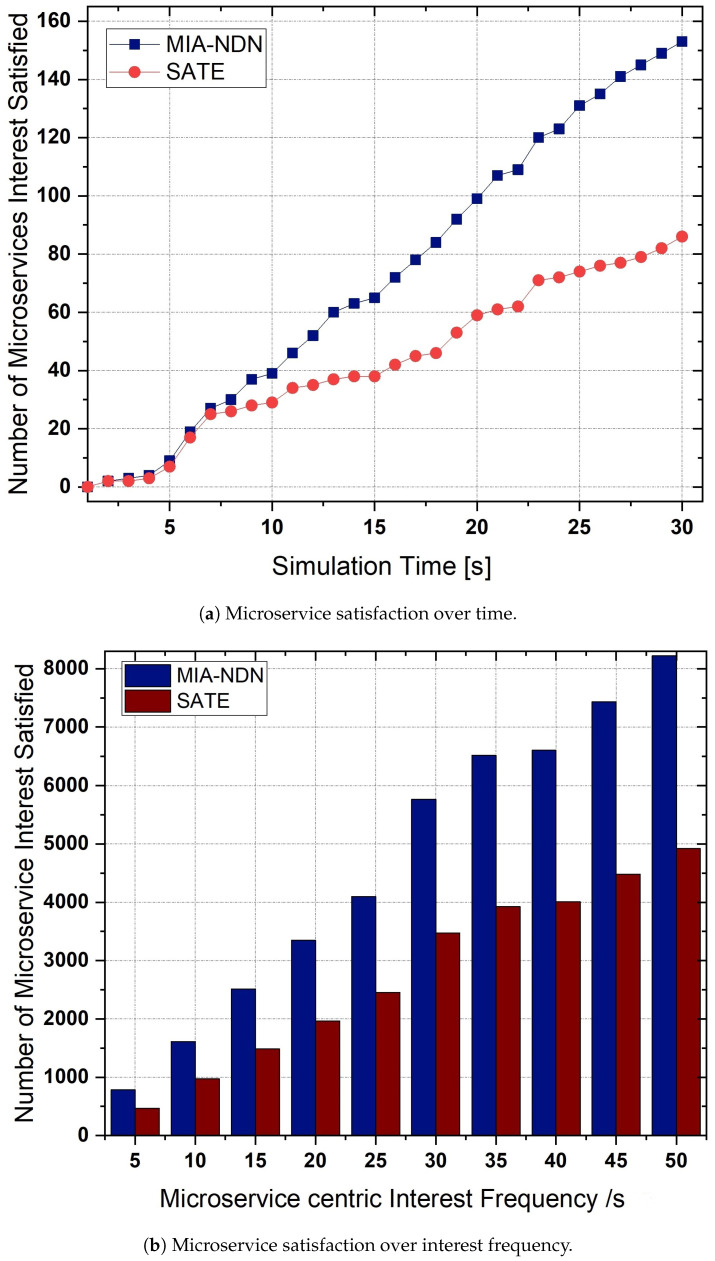
Microservice interest satisfaction as a function of microservice interest frequency.

**Figure 9:**
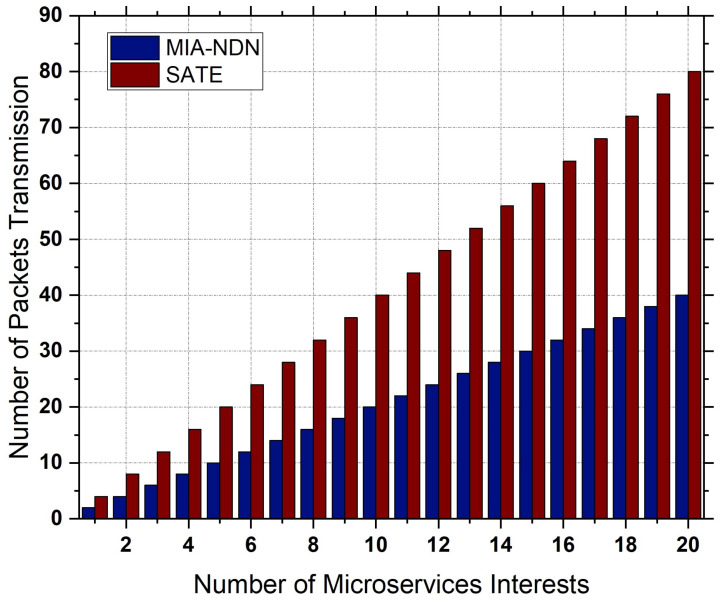
Total number of packet transmissions against the number of microservices.

**Figure 10:**
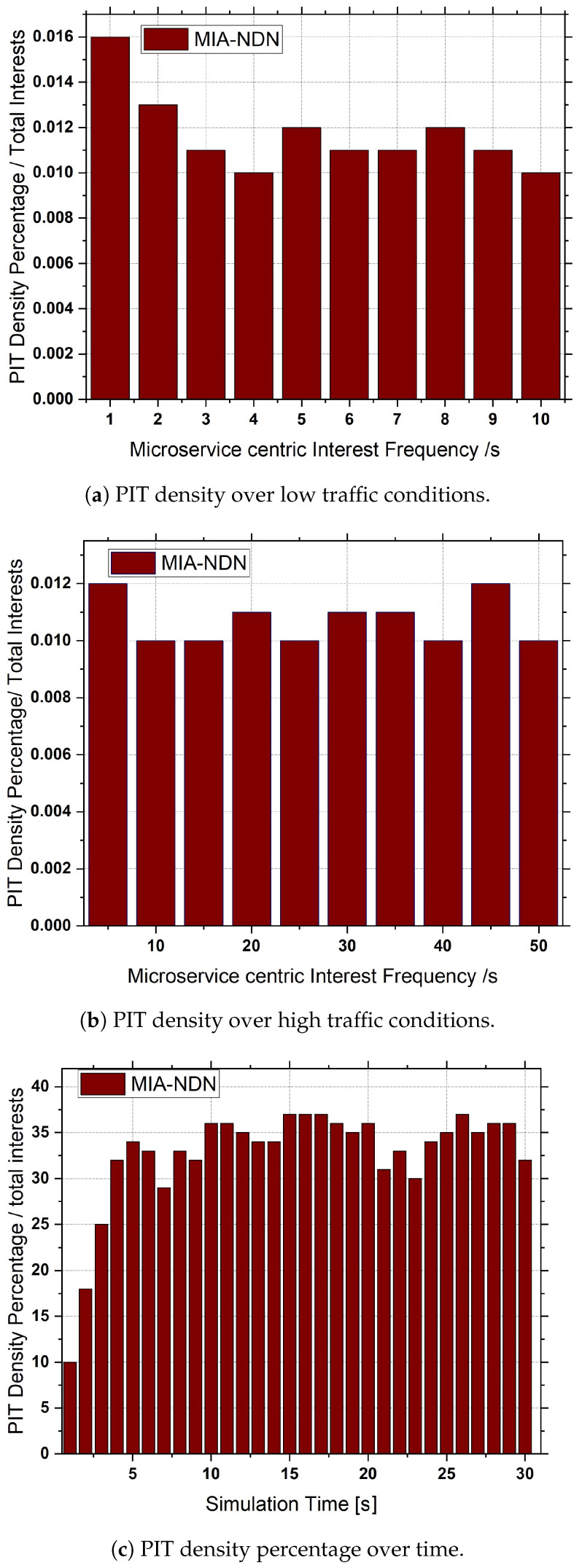
Microservice interest satisfaction as a function of microservice interest frequency.

1. **Interest aggregation:** The microservice-centric interest aggregation as a function of the microservice-based interest frequency is depicted in Figure 7a,b. We varied the interest frequency to analyze the interest aggregation for both low (e.g., 1 to 10 interests/s) traffic scenarios as shown in Figure 7a and high traffic conditions (i.e., 10 to 50 interests/s) shown in Figure 7b.
  The results shown in the figures indicate that, in both traffic conditions, MIA-NDN had less aggregation compared to the benchmark scheme. The rationale is that MIA-NDN incorporates the microservice input parameters in addition to the microservice name in the interest’s aggregation process. If both the microservice-centric interest names and the number of input parameters are the same, MIA-NDN performs interest aggregation. If microservices have the same interest names but a different number of parameters or their corresponding values, MIA NDN considers those interests as unique and avoids false interest aggregation. However, the benchmark scheme ignores the microservice input parameters as well as their corresponding values resulting in high packet aggregation (i.e., false aggregation). The false aggregated microservice interests fail to return the computation results, which turn into network resource wastage, increased latency, and congestion in the network.
2. **Microservice satisfaction:** MIA-NDN evaluated the performance in terms of microservice satisfaction at different time intervals as well as against the microservice-centric interest frequency, as shown in Figure 8a and Figure 8b respectively.
  The simulation results in both scenarios revealed that MIA-NDN outperformed the serving-at-edge schemes in satisfying the microservice-centric heterogeneous computation requests. The reason is that MIA-NDN includes the microservice parameters as well as their corresponding values in the hash generation and process and inserts the generated hash in the PIT table. The aggregated hash is utilized to check the already existing same-named entry in the PIT upon a new interest packet reception. Interest aggregation is performed if the same hash value is found, otherwise the interest packet is considered a unique packet and the corresponding forwarding is performed to fetch the data. The whole procedure avoids false aggregation and increases the microservices satisfaction ratio. It is clear from the results in both cases that MIA-NDN highly reduces the false aggregation and enhances the microservices satisfaction ratio. In contrast, the benchmark scheme performs false packet aggregation due to a lack of consideration of microservice parameters and their corresponding values, resulting in a low microservice satisfaction ratio.
3. **Transmission overhead:**Figure 9 shows the transmission overhead as a function of microservice-centric interest frequency.
  To analyze the transmission overhead, we vary the microservices request rate between 1 interest/s to 20 interests/s. From the figure, it can be observed that MIA-NDN has a lower transmission overhead compared to the benchmark scheme. The main reason behind this is that the MIA-NDN scheme generates only two packets against one microservice computation request, e.g., (i) a microservice computation interest toward the compute node, and (ii) the computed result data packet from the compute node. Contrarily, the benchmark scheme generates a higher number of packets to perform a microservice computation, e.g., computing interest requests, acknowledgment packet from the computing node, data packet, and acknowledgment packet from the consumer node. The large number of packets generated by the benchmark scheme to deliver the computed results produce high transmission overhead as depicted in Figure 9. However, MIA-NDN has low transmission overhead compared with the benchmark scheme as only a single data packet is generated against the consumer request.
4. **PIT density analysis:** We analyze how densely the MIA-NDN populates the PIT table at both high and low traffic conditions by varying the number of microservice-centric computation interest packets, as shown in Figure 10a,b.
  We also analyzed the PIT density at different time intervals as shown in Figure 10c. The results clearly show that MIA-NDN maintains fewer entries in the PIT table and enables more computation requests to be accommodated in the PIT table. The rationale is that the MIA-NDN dynamic PIT lifetime calculation strategy evacuates the PIT entry upon computation result retrieval and enables the incoming request to be inserted in the PIT table. Therefore, in both high and low traffic conditions, the MIA-NDN occupies less in PIT. Moreover, in Figure 10c, we analyze the number of entries in the PIT table at different timer intervals (1 s to 30 s) with a request rate of 10 interests/s. The results clearly show that MIA-NDN has a smaller number of entries in the PIT table due to the provided dynamic PIT entry lifetime management mechanism.

## 5. Conclusions and Future Work

In this paper, we propose an MIA-NDN scheme to enable microservice-centric communication in the NDN architecture. In this regard, the proposed scheme designed (i) a microservice-centric interest-naming structure, (ii) input parametric aware interest aggregation based on the hashing mechanism, and (iii) a dynamic PIT timer calculation and allocation to achieve efficient microservice computation and communication. The simulation results demonstrate the superiority of our scheme against the benchmark work. MIA-NDN showed significant supremacy in terms of same-name microservice computation requests with input parameter level aggregations, a microservice computation request satisfaction rate, and reduced network overhead. Further, to evaluate the scalability of the proposed scheme, we analyzed the PIT table-maintained entries based on low and high traffic as well as different time interval scenarios, and found that our scheme maintains a low percentage of entries in the PIT table.

In future work, we will aim to analyze the computational complexity of the proposed scheme and design microservice mapping and microservices migration to provide computation resources closer to the data source by employing artificial intelligence techniques to achieve maximal node resource utilization and guarantee the QoS of delay-sensitive and compute-intensive application tasks.

## Figures and Tables

**Table 1:** Simulation parameters.

Parameter	Value
Simulator	NS3 (NDNSim)
Communication Stack	NDN
Environment	802.3
Total number of nodes	10
Edge nodes	2
Consumers	2
NDN Routers	6
PIT Time	Dynamic
Topology	(Figure 2)
Simulation time	300 s

## Data Availability

Not applicable.
